# Editorial: *Clostridioides difficile* infection

**DOI:** 10.3389/fmed.2024.1372813

**Published:** 2024-02-26

**Authors:** Guido Granata, Frédéric Barbut, Nicola Petrosillo

**Affiliations:** ^1^Systemic and Immune Depression-Associated Infection Unit, National Institute for Infectious Diseases “L. Spallanzani”, IRCCS, Rome, Italy; ^2^National Reference Laboratory for Clostridioides Difficile, Hôpital Saint Antoine, Assistance Publique-Hôpitaux de Paris, Paris, France; ^3^Infection Prevention and Control Service, Fondazione Policlinico Universitario Campus Bio-Medico, Rome, Italy

**Keywords:** *Clostridioides difficile* infection, COVID-19 pandemic, delayed CDI diagnosis, misdiagnosis, CDI underdiagnosis, community CDI, companion animals and *Clostridioides difficile*, *Clostridiodes difficile* asymptomatic carriers

*Clostridioides difficile* (CD) is a Gram-positive, anaerobic bacterium that is the leading cause of nosocomial diarrhea worldwide. CD contributes to increased morbidity, mortality, and prolonged hospitalization (1, 2). Although research has advanced our understanding of the epidemiology and clinical management of *Clostridioides difficile infection* (CDI), there is still a substantial need for studies to elucidate critical aspects of this disease.

This Research Topic includes five original research articles and two review articles that explore specific aspects of CDI. The articles cover CDI incidence among COVID-19 patients, factors that contribute to misdiagnosis and underdiagnosis of CDI, the role of human and non-human reservoirs in the pathogen's transmission chain within hospitals and communities, and the latest approaches for CDI treatment.

In recent years, significant progress has been made in understanding the pathogenesis of CDI and the role of gut microbiota during CDI development (3).

CD is transmitted through the fecal-oral route in the form of spores. The presence of primary bile acids in the small intestine has been recognized as one of the factor controlling the germination of CD spores (4). The destruction of microbiota, usually caused by antibiotics, can create conditions that favor colonization and growth of vegetative CD cells.

Indeed, bile acids are a significant factor in the control of CD proliferation and CDI development (5, 6).

Modification of bile acids by microbiota contributes to host resistance against intestinal pathogens, such as CD. The bacteria *C. scindens* has a role in this process by converting primary bile acids to secondary bile acids, which inhibits the growth of CD (6).

After broad-spectrum antibiotic treatment, the patient's intestinal microflora is disrupted, leading to a reduction in the relative abundance of *C. scindens*. The imbalance between the intestinal colonization of CD and *C. scindens* prevents the metabolism of bile acids, resulting in an increase in the ratio of primary to secondary bile acids. This, in turn, facilitates the germination and overgrowth of CD (6).

Even after adequate antibiotic therapy against CD, CDI recurrences are observed due to the germination of remaining CD spores, favored by the relative increase in primary bile acids (5, 6).

Moreover, recent studies suggest that *Enterococci* and CD may interact through metabolic cross-talk during CDI, enhancing mutual colonization, persistence, and pathogenesis in the gut (7). *Enterococci* can increase toxin production, thereby enhancing the pathogenesis of CD (7). Additionally, the biofilm structure of *Enterococcus faecalis* plays a role in enhancing the survival of CD following antibiotics exposure (8).

Vegetative CD cells possess genes encoding up to three different toxins, which cause the onset of clinical symptoms. Toxin A and toxin B are glucosyltransferases that bind to host cell receptors, are endocytosed by host cells, and subsequently inactivate Rho family GTPases by glycosylation (9). This inactivation destroys the host cytoskeleton and accelerates the breakdown of epithelial barrier function. A third toxin, called CD transferase or binary toxin, is an ADP-ribosyltransferase. Upon endocytosis, the binary toxin catalyzes the depolymerization of actin (9). Together, the CD toxins can disrupt the colonic epithelium to cause fluid secretion, inflammation and tissue damage.

Finally, the long-term impact of SARS-CoV-2 and CD coinfection remains unknown. Several mechanisms can influence the onset and clinical course of CDI in SARS-CoV-2 infected patients. The gastrointestinal tract expresses SARS-CoV-2 receptors such as the angiotensin-converting enzyme 2 and transmembrane serine protease 2 (10). Therefore, COVID-19 can directly damage the gastrointestinal tract.

The effects of SARS-CoV-2 virus replication include disruption of both the innate and adaptive immune response, damage to the host gastrointestinal barrier, and detrimental effects on the gut microbiome (11–14). Delayed diagnosis of CDI may occur due to misinterpretation of gastrointestinal symptoms in COVID-19 patients, which are mistakenly attributed to the action of the SARS-CoV-2 virus rather than CD (11–14).

The epidemiology and clinical spectrum of CDI appear to have undergone changes during the COVID-19 pandemic, requiring further clarification. Tools are needed to promptly identify CDI among COVID-19 patients, particularly those at high risk of severe CDI (15).

There is limited data on the incidence and features of CDI in COVID-19 patients. However, most available studies have not reported a significant increase in CDI incidence during the COVID-19 pandemic compared to the pre-pandemic period (16–18).

In a retrospective study, Vázquez-Cuesta et al. compared the incidence of hospital-acquired CDI between hospitalized patients with and without COVID-19. The aim was to delineate the characteristics of COVID-19 patients who developed CDI. In 2020, they identified 68 episodes of hospital-acquired CDI in COVID-19 patients (14.75/10,000 days) and 159 in non-COVID-19 patients (5.54/10,000 days). In contrast, the incidence during the pre-pandemic period in 2019 was 6.80/10,000 days. The study found that hospital-acquired CDI is more common among COVID-19 patients than non-COVID-19 patients. It is worth noting that COVID-19-associated hospital-acquired CDI cases had longer hospital stays (*p* < 0.05) (Vázquez-Cuesta et al.). Moreover, COVID-19 patients with hospital-acquired CDI had a significantly higher in-hospital mortality rate (23.1%) compared to patients with hospital-acquired CDI but without COVID-19 (14.3%) (*p*: 0.006). The authors suggest that the higher occurrence of hospital-acquired CDI in COVID-19 patients could be due to risk factors present in this population, such as prolonged hospitalization and extensive antibiotic use (Vázquez-Cuesta et al.).

Furthermore, Azimirad et al. conducted a literature review on potential risk factors contributing to the increased incidence of CDI in COVID-19 patients. The review suggests that hospitalized elderly patients under antibiotic treatment may be more susceptible to CDI. The use of broad-spectrum antibiotics, such as cephalosporins or fluoroquinolones, can affect the gut microbiota of COVID-19 patients. This can reduce colonization resistance against opportunistic pathogens like CD, potentially leading to CDI. Additionally, patients with CDI may have prolonged presence of SARS-CoV-2 viral particles in their feces for up to 1 month (19). This coinfection could increase the nosocomial transmission of both SARS-CoV-2 and CD through fecal materials (19). The review highlights the importance of infection prevention measures and judicious antibiotic use in managing COVID-19 patients (Azimirad et al.).

Currently, the rates of underdiagnosis and misdiagnosis of CDI remain high. Diagnostic algorithms typically involve a two-step protocol that uses stool enzyme immunoassays to detect the CD glutamate dehydrogenase antigen and CD toxins. The presence of CD toxins in the patient's stool is crucial for supporting the diagnosis of CDI. However, recent research suggests that CD strains with low toxin production, which are found in toxin-negative clinical stool specimens, can still cause disease in a rodent CDI model (20).

To address this concern, Anwar et al. conducted a longitudinal surveillance study to determine the frequency of discrepant CDI specimens, specifically those that are GDH positive but toxin negative. The study evaluated 8,910 CD isolates recovered from stool specimens, of which 19.4% (1,733 out of 8,910) were found to harbor CD. It is noteworthy that 1,041 out of the 1,733 CD specimens were toxin-negative by enzyme immunoassays. Among these toxin-negative specimens, 69% (439 out of 636 ribotyped specimens) were found to harbor toxigenic CD strains through DNA ribotyping. Although these strains produced less toxin than those from non-discrepant specimens of the same ribotype, the findings highlight the potential clinical significance of diagnostically discrepant specimens. To prevent misdiagnosis of CDI a thorough re-evaluation of the clinical significance of such specimens is recommended, particularly when there is a strong clinical suspicion of CDI (Anwar et al.).

Furthermore, asymptomatic carriers of CD may serve as a significant reservoir, contributing to the transmission of CD within healthcare settings. A comprehensive national, multicenter, point prevalence study was conducted across 11 French hospitals to estimate the rate of toxigenic CD asymptomatic carriage and identify associated risk factors. The study involved 2,389 patients with a median age of 62 years (Jolivet et al.).

Of the patients, 185 (7.7%) tested positive for CD, including 93 (3.9%) with toxigenic strains. It is noteworthy that 77 (82.8%) of the toxigenic carriers were asymptomatic, resulting in a prevalence of asymptomatic CD carriers patients of 3.2%. Two risk factors that were positively associated with asymptomatic carriage of toxigenic CD: co-carriage of multidrug-resistant organisms (adjusted Odds Ratio: 2.3, 95% confidence interval: 1.2–4.7, *p*: 0.02) and a history of previous CDI episodes (adjusted Odds Ratio: 5.8, 95% confidence interval: 1.2–28.6, *p*: 0.03). The study found that the consumption of raw milk was protective against toxigenic CD colonization (adjusted Odds Ratio: 0.5, 95% confidence interval: 0.2–0.9, *p*: 0.01). This may be due to the barrier effect provided by bacteria associated with raw milk (Jolivet et al.).

The epidemiology of CD is changing, with a significant increase in community-acquired CDI. It is important to understand both in-hospital and community transmission routes to develop appropriate interventions.

Alves et al. conducted a study to identify CD strains in canine and feline populations and assess their genetic similarity to human strains, in order to detect potential interspecies transmission. In this study, 335 fecal samples were collected from dogs and 140 from cats in Portugal. The CD isolates were characterized by toxigenic profile and ribotyping. Antimicrobial resistance determinants were examined in resistant isolates, and whole-genome sequencing was performed on 83 strains from animals and humans. The CD positivity rate was found to be 23.1% (110/475), with toxigenic strains constituting 50% of animal carriage (Alves et al.). The study found that 17.1% of isolates were resistant to metronidazole and 12.4% were resistant to moxifloxacin due to the “pCD-METRO” plasmid and point mutations in the gyrA and rpoB genes, respectively. This study confirmed that toxigenic CD can be transmitted from companion animals to humans. Genetic analysis has identified clusters that integrate isolates from both animal and human sources, supporting the potential for clonal interspecies transmission or shared environmental contamination (Alves et al.).

Recurrent CDI is a persistent challenge despite existing therapies. To address this issue, non-antimicrobial strategies such as monoclonal anti-toxin antibodies, fecal microbiota transplantation, live bacterial products, and CD vaccines have been explored. However, further studies are needed to confirm their efficacy and safety.

Barbosa et al. conducted a literature review on the use of probiotics to prevent CDI. The authors identified seven studies on the issue, but no consensus was reached among the included studies. Three studies supported the use of probiotics for reducing hospital-onset CDI in elderly patients, while four studies found no significant benefit. The authors concluded that more research is necessary, and current evidence does not adequately support the prescription of probiotics for CDI prevention (Barbosa et al.).

It has been shown that fecal microbiota transplantation is a promising approach. A cost-effectiveness study was conducted to compare different treatments for recurrent CDI (Lan et al.). The study utilized a Markov model with a one-year time horizon and was based on data from a large Taiwanese hospital. The results showed that fecal microbiota transplantation was more cost-effective than vancomycin, providing gains in quality-adjusted life years. Compared to fidaxomicin, fecal microbiota transplantation was also found to be cost-effective, although the improvement in quality of life for patients with inflammatory bowel disease who have recurrent CDI was only marginal (Lan et al.).

## Author contributions

GG: Conceptualization, Writing – original draft, Writing – review & editing, Supervision. FB: Conceptualization, Supervision, Writing – original draft, Writing – review & editing. NP: Conceptualization, Supervision, Writing – original draft, Writing – review & editing.

## References

[1] Magill SS O'Leary E Janelle SJ Thompson DL Dumyati G Nadle J. Emerging infections program hospital prevalence survey team. Changes in prevalence of health care-associated infections in US hospitals. N Engl J Med. (2018) 379:1732–44. 10.1056/NEJMoa1801550 30380384 PMC7978499

[2] Suetens C Latour K Kärki T Ricchizzi E Kinross P Moro ML. Healthcare-Associated Infections Prevalence Study Group. Prevalence of healthcare-associated infections, estimated incidence and composite antimicrobial resistance index in acute care hospitals and long-term care facilities: results from two European point prevalence surveys, 2016 to 2017. Euro Surveill. (2018) 23:1800516. 10.2807/1560-7917.ES.2018.23.46.1800516 30458912 PMC6247459

[3] Cataldo MA Granata G Petrosillo N. Clostridium difficile infection: new approaches to prevention, non-antimicrobial treatment, and stewardship. Expert Rev Anti Infect Ther. (2017) 15:1027–40. 10.1080/14787210.2017.1387535 28980505

[4] Shen A. *Clostridioides difficile* spore formation and germination: new insights and opportunities for intervention. Annu Rev Microbiol. (2020) 74:545–66. 10.1146/annurev-micro-011320-011321 32905755

[5] Ng KM Ferreyra JA Higginbottom SK Lynch JB Kashyap PC Gopinath S. Microbiota-liberated host sugars facilitate post-antibiotic expansion of enteric pathogens. Nature. (2013) 502:96–9. 10.1038/nature12503 23995682 PMC3825626

[6] Buffie CG Pamer G. Microbiota-mediated colonization resistance against intestinal pathogens. Nat Rev Immunol. (2013) 13:790–801. 10.1038/nri3535 24096337 PMC4194195

[7] Smith AB Jenior ML Keenan O Hart JL Specker J Abbas A et al. Enterococci enhance *Clostridioides difficile* pathogenesis. Nature. (2022) 611:780–6. 10.1038/s41586-022-05438-x 36385534 PMC9691601

[8] Granata G Schiavone F Taglietti F Petrosillo N. *Clostridioides difficile* and enterococci's interplay in the human gut: bacterial alliance or competition? A systematic literature review. J. Clini. Med. (2023) 12:4997. 10.3390/jcm12154997 PMC10420055 37568399

[9] Kordus SL Thomas AK Lacy DB. *Clostridioides difficile* toxins: mechanisms of action and antitoxin therapeutics. Nat Rev Microbiol. (2022) 20:285–98. 10.1038/s41579-021-00660-2 34837014 PMC9018519

[10] Cao W Li T. COVID-19: towards understanding of pathogenesis. Cell Res. (2020) 30:367–9. 10.1038/s41422-020-0327-4 32346073 PMC7186532

[11] Granata G Petrosillo N Adamoli L Bartoletti M Bartoloni A Basile G., On behalf of the ReCloDi recurrence Of *Clostridioides difficile* infection study group. prospective study on incidence, risk factors and outcome of recurrent *Clostridioides difficile* infections. J Clin Med. (2021) 10:1127. 10.3390/jcm10051127 33800334 PMC7962640

[12] Blanco-Melo D Nilsson-Payant BE Liu WC Uhl S Hoagland D Møller R. Imbalanced Host Response to SARS-CoV-2 Drives Development of COVID-19. Cell. (2020) 181:1036–45. 10.1016/j.cell.2020.04.026 32416070 PMC7227586

[13] Cha MH Regueiro M Sandhu DS. Gastrointestinal and hepatic manifestations of COVID-19: a comprehensive review. World J Gastroenterol. (2020) 26:2323–32. 10.3748/wjg.v26.i19.2323 32476796 PMC7243653

[14] Zuo T Zhang F Lui GCY Yeoh YK Li AYL Zhan H. Alterations in gut microbiota of patients with COVID-19 during time of hospitalization. Gastroenterology. (2020) 159:944–55. 10.1053/j.gastro.2020.05.048 32442562 PMC7237927

[15] Lakkasani S Chan KH Shaaban HS. *Clostridiodes difficile* in COVID-19 patients, Detroit, Michigan, USA, March-April 2020. Emerg Infect Dis. (2020) 26:2299–300. 10.3201/eid2609.202505 32620183 PMC7454088

[16] Granata G Petrosillo N Al Moghazi S Caraffa E Puro V Tillotson G. The burden of *Clostridioides difficile* infection in COVID-19 patients: a systematic review and meta-analysis. Anaerobe. (2022) 74:102484. 10.1016/j.anaerobe.2021.102484 34843959 PMC8616765

[17] Granata G Bartoloni A Codeluppi M Contadini I Cristini F Fantoni M. On behalf Of the CloVid study group. The burden of *Clostridioides difficile* Infection during the COVID-19 pandemic: a retrospective case-control study in Italian Hospitals (CloVid). J Clin Med. (2020) 9:3855. 10.3390/jcm9123855 33260943 PMC7761275

[18] Weiner-Lastinger LM Pattabiraman V Konnor RY Patel PR Wong E Xu SY. The impact of coronavirus disease 2019 (COVID-19) on healthcare-associated infections in 2020: a summary of data reported to the National Healthcare Safety Network. Infect Control Hosp Epidemiol. (2022) 43:12–25. 10.1017/ice.2021.362 34473013

[19] Khanna S Kraft CS. The interplay of SARS-CoV-2 and *Clostridioides difficile* infection. Future Microbiol. (2021) 16:439–43. 10.2217/fmb-2020-0275 33847139 PMC8054643

[20] Anwar F Roxas BAP Shehab KW Ampel NM Viswanathan VK Vedantam G. Low-toxin *Clostridioides difficile* RT027 strains exhibit robust virulence. Emerg Microbes Infect. (2022) 11:1982–93. 10.1080/22221751.2022.2105260 35880487 PMC9361768

